# Guidelines for Artificial Intelligence in Medicine: Literature Review and Content Analysis of Frameworks

**DOI:** 10.2196/36823

**Published:** 2022-08-25

**Authors:** Norah L Crossnohere, Mohamed Elsaid, Jonathan Paskett, Seuli Bose-Brill, John F P Bridges

**Affiliations:** 1 Department of Biomedical Informatics The Ohio State University College of Medicine Columbus, OH United States; 2 Division of General Internal Medicine Department of Internal Medicine The Ohio State University College of Medicine Columbus, OH United States

**Keywords:** artificial intelligence, translational science, translational research, ethics, engagement, reproducibility, transparency, effectiveness, medicine, health care, AI

## Abstract

**Background:**

Artificial intelligence (AI) is rapidly expanding in medicine despite a lack of consensus on its application and evaluation.

**Objective:**

We sought to identify current frameworks guiding the application and evaluation of AI for predictive analytics in medicine and to describe the content of these frameworks. We also assessed what stages along the AI translational spectrum (ie, AI development, reporting, evaluation, implementation, and surveillance) the content of each framework has been discussed.

**Methods:**

We performed a literature review of frameworks regarding the oversight of AI in medicine. The search included key topics such as “artificial intelligence,” “machine learning,” “guidance as topic,” and “translational science,” and spanned the time period 2014-2022. Documents were included if they provided generalizable guidance regarding the use or evaluation of AI in medicine. Included frameworks are summarized descriptively and were subjected to content analysis. A novel evaluation matrix was developed and applied to appraise the frameworks’ coverage of content areas across translational stages.

**Results:**

Fourteen frameworks are featured in the review, including six frameworks that provide descriptive guidance and eight that provide reporting checklists for medical applications of AI. Content analysis revealed five considerations related to the oversight of AI in medicine across frameworks: transparency, reproducibility, ethics, effectiveness, and engagement. All frameworks include discussions regarding transparency, reproducibility, ethics, and effectiveness, while only half of the frameworks discuss engagement. The evaluation matrix revealed that frameworks were most likely to report AI considerations for the translational stage of development and were least likely to report considerations for the translational stage of surveillance.

**Conclusions:**

Existing frameworks for the application and evaluation of AI in medicine notably offer less input on the role of engagement in oversight and regarding the translational stage of surveillance. Identifying and optimizing strategies for engagement are essential to ensure that AI can meaningfully benefit patients and other end users.

## Introduction

Artificial intelligence (AI) allows computers to accomplish tasks that normally require the use of human intelligence. Creating AI, or an AI computer system, begins when developers feed the system existing data and allow it to “learn.” This learning experience enables AI to understand, infer, communicate, and make decisions similar to, or better than, humans [1,2]. The use of AI in medicine is an area of rapid growth, with worldwide spending on health care AI technologies estimated to reach US $45 billion by 2026 [3]. AI is used across numerous medical specialties, and can be applied to inform medical decision-making in numerous ways, such as through expediting and reducing the costs of drug discovery [4]; offering insight that aids clinicians in diagnosing, prognosing, or optimizing treatment plans at the point of care; and automating medical administration activities such as appointment reminders [5].

Numerous concerns have been raised regarding a lack of oversight for the rapid development and expansion of AI in medicine. Commentators have drawn attention to the potential weaknesses and limitations of AI in medicine, including challenges spanning ethical, legal, regulatory, methodological, and technical domains [6]. These perspectives have highlighted pitfalls such as implicit bias, reproducibility, and clinical validity [7-9]. There is further concern that the methods for development and approaches for evaluation of AI are not as robust and rigorous as those of other medical interventions [10]. Although several best practices for the design, implementation, and evaluation of AI can be informed by the biostatistical and data science literature, such guidelines are not sufficient to address all concerns related to AI in medicine [11].

Translational science is the study of how to turn concepts, observations, or theories into actions and interventions by following defined stages of research and development. This is done to improve the health of individuals and society [12]. The stages of the translation for typical diagnostics and therapeutics often follow a traditional pathway from ideation to community implementation and social benefit [13]. Very clear, albeit complex, translation pathways exist for diagnostics and therapeutics, and are enforced by regulatory, funding, and ethical review. For AI, the translational pathway is less well-defined and overseen, but generally includes stages such as development, design, validation, reporting, implementation, and scaling [14]. Nevertheless, questions remain regarding how to adapt translational oversight mechanisms for AI in medicine [15].

Developing robust guidance for the oversight of AI along its translational pathway is essential to facilitating its clinical impact [16]. Several professional organizations have developed frameworks to address concepts specific to the development, reporting, and validation of AI in medicine [2,16-20]. These frameworks are focused primarily on informing the technological developers of AI (such as by offering guidance on how to promote transparency in the design and reporting of AI algorithms), rather than informing the clinical application of AI [2,20]. Regulatory oversight of AI is also in nascent stages. Guidance on how to critically evaluate actual applications of AI in medicine are currently in development by the US Food and Drug Administration (FDA) [21]. The European Commission has led a multidisciplinary initiative to increase the trustworthiness of AI [22,23], and the European Medicines Agency has identified the regulation of AI as a strategic priority [24].

Identifying considerations for the oversight of AI across the translational spectrum is essential to increasing the utility of AI in medicine. In this study, we explored and characterized existing frameworks regarding the oversight of AI in medicine. We then identified specific considerations raised in these frameworks and mapped them to different stages of the translational process for AI.

## Methods

### Identification of Frameworks

We performed a literature review to identify guidance on the use of predictive analytic AI in medicine. The search spanned the PubMed, Web of Science, and Embase databases, and also included a grey literature search of Google. Key terms for searching included “artificial intelligence,” “machine learning,” “guidance as topic,” and “translational science.” Documents were included if they provided generalized guidance (ie, were a framework) on applying or evaluating AI in medicine. Documents that described specific AI applications without offering overarching guidance on the use of AI were excluded. The reference lists of included frameworks were screened for additional relevant sources. Frameworks were not restricted to the use of AI in any specific condition or medical setting. The time period of the review was January 2014 to May 2022; 2014 was selected as the cut-off point, as this was the year when regulatory agencies in the United States and Europe began using the authorization designation of “software as a medical device,” which includes regulation over AI.

### Data Abstraction, Coding, and Analysis

A structured abstraction process was used to collect general information about each framework, including title, author/affiliation, year, summary, and intended audience. Frameworks were analyzed using content analysis, which is an approach for exploring themes or patterns from textual documents [25]. Content analysis of text-based sources can be either qualitative, where theory or themes are identified, or quantitative, wherein numeric information is derived [26]. We employed both approaches in this study. We first used qualitative content analysis to identify the different topics (“domains”) discussed by frameworks. Codes for these domains were not developed a priori but were rather identified inductively through a reading of the frameworks. Frameworks were evaluated to assess whether they discussed each domain in relation to each of the translational stages [27]. Stages of AI translation were predefined to reflect the full AI product lifecycle, including development, validation, reporting, implementation, and surveillance. We used evaluation matrix methodologies [28-30] to depict how many frameworks described the domains identified through content analysis.

Data were visualized using several approaches. First, we used spider plots to visualize, for each individual framework, how many stages of translation were discussed in relation to each of the five domains. Second, we applied a heatmap to depict the number of frameworks discussing a given domain across each translational stage. The heatmap cross-walked the domains across the five stages of translation.

## Results

### Overview of the Frameworks

A total of 14 documents were included in the review, which are summarized in Table 1. One framework was published in 2016 (*Guidelines for Developing and Reporting* [31]) and all others were published from 2019 to 2020. Several of the frameworks were developed through pathways with professional organizations (*AI in Health Care* [32], *CONSORT-AI* [20], *SPIRIT-AI* [2], *DECIDE-AI* [33]). All frameworks were published as journal articles, and *AI in Healthcare* was published as both a journal article [7] and a White Paper [32]; since the journal article was a synopsis of the White Paper, the latter was used as the primary document of reference for this review. The frameworks explored in this review were generally consensus- rather than evidence-based. All but three frameworks [19,34,35] identified greater than one intended audience, and typical audiences included AI developers, investigators, clinicians, patients, and policymakers. Frameworks provided either general guidance on the use of AI in medicine, typically in narrative prose (herein referred to as “descriptive frameworks”) [19,32,34-37] or guidance specifically on the reporting of AI studies in medicine, typically in checklist style (herein referred to as “reporting frameworks”) [2,17,20,31,33,38-40].

**Table 1:** Summary of frameworks for the use of artificial intelligence (AI) in medicine.

Frameworks		Summary	Audience
**Descriptive frameworks**			
	*AI in Healthcare*, Matheny et al [32]^a^	Describes general challenges and opportunities associated with the use of AI in medicine	AI developers, clinicians, patients, policymakers
	*Clinician Checklist*, Scott et al [34]	Describes recommendations on evaluating the suitability of AI applications for clinical settings	Clinicians
	*Ethical Considerations*, Char et al [36]	Describes a roadmap for considering ethical aspects of AI with health care applications	AI developers, investigators, clinicians, policymakers
	*Evaluating AI*, Park et al [37]	Describes an evaluation framework for the application of AI in medicine	Investigators, health care organizations
	*Users’ Guide*, Liu et al [19]	Describes an approach for assessing published literature using AI for medical diagnoses	Clinicians
	*Reporting and Implementing Interventions*, Bates et al [35]	Describes barriers to the implementation of AI in medicine and provides solutions to address them	Health care organizations
**Reporting frameworks**			
	*20 Critical Questions*, Vollmer et al [17]	Proposes 20 questions for evaluating the development and use of AI in research (20 reporting items)	Investigators, clinicians, patients, policymakers
	*Comprehensive Checklist*, Cabitza and Campagner [38]	Proposes a comprehensive checklist for the self-assessment and evaluation of medical papers (30 reporting items)	Investigators, editors and peer reviewers
	*CONSORT*^b^*-AI*, Liu et al [20]^a^	Provides reporting guidelines for clinical trials evaluating interventions with an AI component (25 core and 15 AI-specific reporting items)	AI developers, investigators
	*CAIR*^c^*Checklist*, Olczak et al [39]	Provides guidelines and an associated checklist for the reporting of AI research to clinicians (15 reporting items)	Investigators, developers, clinicians
	*DECIDE-AI,* Vasey et al [33]^a^	Provides reporting guidelines for evaluations of early-stage clinical decision support systems developed using AI (10 generic and 17 AI-specific reporting items)	Investigators, clinicians, patients, policymakers
	*Guidelines for Developing and Reporting*, Luo et al [31]	Provides guidelines for applying and reporting AI model specifications/results in biomedical research (12 reporting items)	AI developers, investigators
	*MINIMAR*^d^, Hernandez-Boussard et al [40]	Provides minimum reporting standards for AI in health care (16 reporting items)	AI developers, investigators
	*SPIRIT*^e^*-AI*, Rivera et al [2]^a^	Provides guidelines for clinical trials protocols evaluating interventions with an AI component (25 core and 15 AI-specific reporting items)	AI developers, investigators

^a^Publication associated with a professional organization; AI in Healthcare=National Academy of Medicine; CONSORT-AI=CONSORT Group; DECIDE-AI=DECIDE-AI Expert Group; SPIRIT-AI=SPIRIT Group.

^b^CONSORT: Consolidated Standards of Reporting Trials.

^c^CAIR: Clinical AI Research.

^d^MINIMAR: Minimum Information for Medical AI Reporting.

^e^SPIRIT: Standard Protocol Items: Recommendations for Interventional Trials.

### Descriptive Frameworks

#### AI in Health Care

Matheny and colleagues [32] synthesized current knowledge related to the accountable development, application, and maintenance of AI in health care. This narrative describes existing and upcoming Al solutions, and underscores current challenges, limitations, and best practices for AI development, implementation, and maintenance.

#### Clinician Checklist

Scott and colleagues [34] proposed a checklist to evaluate the potential impact on clinical decision-making and patient outcomes of emerging machine-learning algorithms. Targeted toward clinicians, the checklist has been tailored for nonexperts, and provides a brief background of relevant machine-learning concepts and examples. The checklist addresses issues such as validity, utility, feasibility, safety, and ethical use.

#### Ethical Considerations

Char and colleagues [36] outlined a systematic approach for addressing ethical concerns surrounding machine-learning health care applications, and highlighted the need for interdisciplinary collaboration of diverse stakeholders. Evaluation and oversight tasks are described at each stage of the machine-learning pipeline from conception to implementation. Key questions and ethical considerations address common concerns found through a literature search as well as considerations that have received less attention.

#### Evaluating AI

Park and colleagues [37] highlighted the need for real-word evaluations of AI applications in health care. They present the phases of clinical trials for drugs and medical devices along with how AI applications could be evaluated in a similar manner. For each phase (including discovery and invention, technical performance and safety, efficacy and side effects, therapeutic efficacy, and safety and effectiveness), they propose appropriate study designs and methods for AI evaluation.

#### Users’ Guide

Liu and colleagues [19] presented a users’ guide to inform primarily clinicians about the major principles of machine learning. They describe the need for effective machine-learning model validation, review basic machine learning concepts, and provide recommendations on effective ways to implement machine-learning models in clinical medicine.

#### Reporting and Implementing Interventions

After presenting clinical examples of beneficial AI use, Bates and colleagues [35] discuss three major bottlenecks slowing the adoption of AI and machine-learning technologies in health care: methodological issues in evaluating AI-based interventions, the need for standards in reporting, and institution hurdles. They also highlight the role of FDA regulation and consider the need for rapid innovation in AI development.

### Reporting Frameworks

#### 20 Critical Questions

Vollmer and colleagues [17] provided a set of 20 questions focused on improving the transparency, replicability, ethics, and effectiveness of AI methods in health care. Statutory regulators and members of national advisory bodies and academic organizations, mostly from the United Kingdom and United States, collaboratively developed the questions.

#### Comprehensive Checklist

Cabitza and Campagner [38] proposed an extensive 30-item checklist to assess the quality of medical machine-learning studies. The checklist has been formatted both for authors to evaluate their own contributions and for reviewers to indicate where revisions may be necessary, and is organized in six phases: problem understanding, data understanding, data preparation, modeling, validation, and deployment.

#### CONSORT-AI

Liu and colleagues [20] extended the CONSORT (Consolidated Standards for Reporting Trials) framework to include additional considerations for the reporting of AI trials. The primary purpose of the extension is to facilitate the transparent reporting of interventional trials using AI, and the reporting checklist also provides some guidance for the development and critical appraisal of AI intervention studies.

#### CAIR Checklist

Olczak and colleagues [39] proposed a checklist for reporting medical AI research to clinicians and other stakeholders. They describe common performance and outcome measures that clinicians should be familiar with, and incorporate guidance about which metrics should be presented at each stage of a manuscript into the checklist. They also address ethical considerations that arise from AI use in health care.

#### DECIDE-AI

Vasey and colleagues [33] presented reporting guidelines for early-stage clinical trials of AI decision-support systems. The checklist focuses on four key aspects: proof of clinical utility, safety, the evaluation of human factors, and preparation for larger trials. This checklist was developed through a consensus process involving 151 experts and 20 stakeholder groups.

#### Guidelines for Developing and Reporting

Luo and colleagues [31] generated a set of guidelines on reporting machine-learning predictive models in biomedical research. The objective of these guidelines is to provide best practices for AI in biomedical research. This framework includes a list of minimum reporting items to be included in research manuscripts and a set of recommendations for optimal use of predictive models.

#### MINIMAR

Hernandez-Boussard and colleagues [40] proposed a list of minimum information that should be reported for all medical AI technologies. This list is intended to promote broader discussion and help inform extensions to other checklists. The four essential components in their guidelines include study population and setting, patient demographic characteristics, model architecture, and model evaluation.

#### SPIRIT-AI

Rivera and colleagues [2] presented reporting guidelines to evaluate clinical trial protocols involving interventions with an AI component. The purpose of the guidelines is to promote transparency and comprehensiveness for clinical trials with AI interventions. The guidelines were developed as AI extensions to the SPIRIT (Standard Protocol Items: Recommendations for Interventional Trials) and CONSORT guidelines.

### Content Domains

#### Overview of Domains

**Figure 1:**
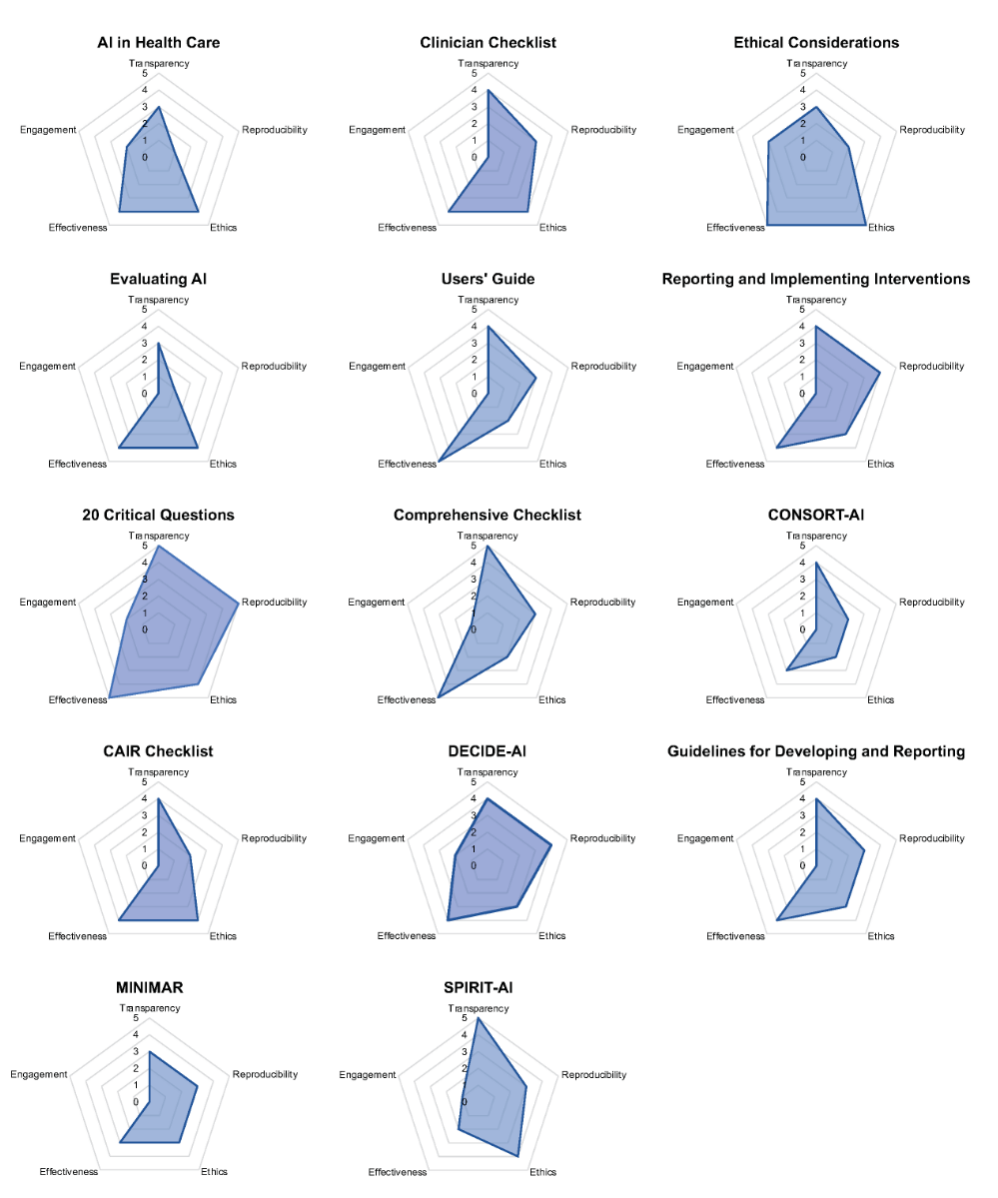
Coverage of frameworks across content domains. AI: artificial intelligence; CAIR: Clinical AI Research; CONSORT: Consolidated Standards of Reporting Trials; MINIMAR: Minimum Information for Medical AI Reporting; SPIRIT: Standard Protocol Items: Recommendations for Interventional Trials.

**Figure 2:**
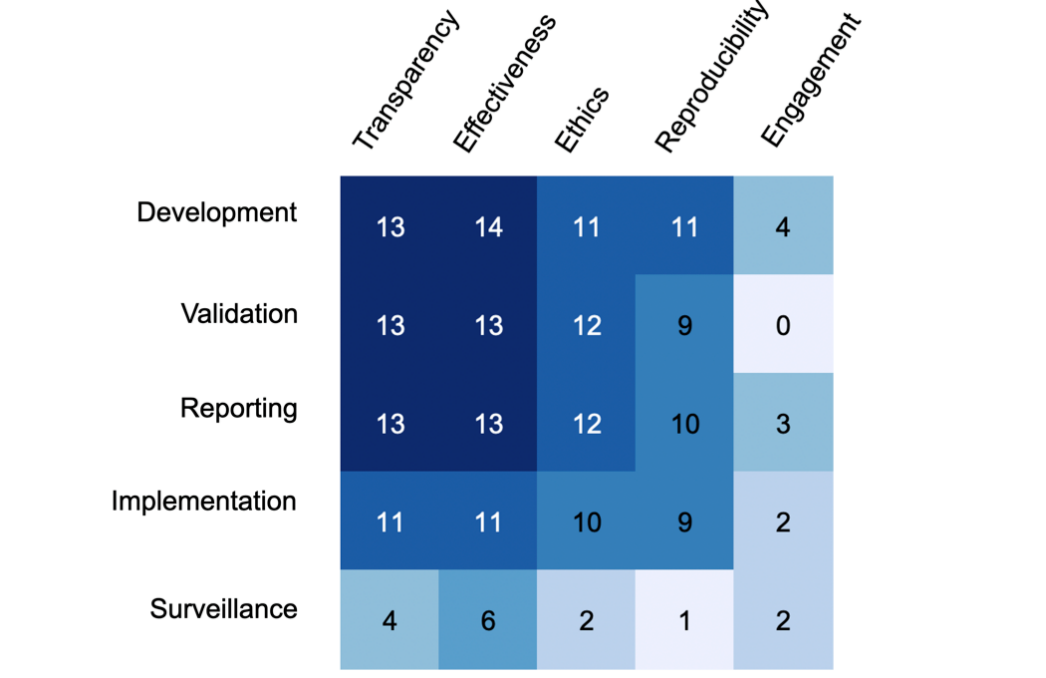
Heatmap of the frameworks' coverage across the five stages of translation. Darker boxes indicate areas where more frameworks offered guidance, whereas lighter boxes indicate areas where fewer frameworks offered guidance.

We identified five domains through the content analysis, including transparency, reproducibility, ethics, effectiveness, and engagement. These domains are described in turn below. Table 2 depicts each framework’s coverage of content domains across translational stages. Figure 1 depicts the coverage of each individual framework and Figure 2 presents the aggregate coverage of frameworks as a heatmap.

**Table 2:** Coverage of frameworks across content domains and translational stages.

Domain and stage			Descriptive frameworks							Reporting frameworks							
		AI^a^ in health care		Clinician Checklist	Ethical Considerations	Evaluating AI	Users’ Guide	Reporting and Implementing Interventions	20 Critical Questions		Comprehensive Checklist	CONSORT^b^- AI	CAIR^c^ Checklist	DECIDE-AI	Guidelines for Developing and Reporting	MINIMAR^d^	SPIRIT^e^- AI
**Transparency**																	
	Development	✓		✓	✓		✓	✓	✓		✓	✓	✓	✓	✓	✓	✓
	Validation			✓	✓	✓	✓	✓	✓		✓	✓	✓	✓	✓	✓	✓
	Reporting	✓		✓	✓		✓	✓	✓		✓	✓	✓	✓	✓	✓	✓
	Implementation			✓		✓	✓	✓	✓		✓	✓	✓	✓	✓		✓
	Surveillance	✓				✓			✓		✓						
**Reproducibility**																	
	Development				✓		✓	✓	✓		✓	✓	✓	✓	✓	✓	✓
	Validation	✓		✓	✓	✓	✓	✓	✓					✓		✓	
	Reporting			✓				✓	✓		✓	✓	✓	✓	✓	✓	✓
	Implementation			✓			✓	✓	✓		✓	✓		✓	✓		✓
	Surveillance								✓								
**Ethics**																	
	Development	✓		✓	✓	✓		✓	✓				✓	✓	✓	✓	✓
	Validation	✓		✓	✓	✓	✓		✓			✓	✓	✓	✓	✓	✓
	Reporting	✓		✓	✓			✓	✓		✓	✓	✓	✓	✓	✓	✓
	Implementation	✓		✓	✓	✓	✓	✓	✓		✓		✓				✓
	Surveillance				✓	✓											
**Effectiveness**																	
	Development	✓		✓	✓	✓	✓	✓	✓		✓	✓	✓	✓	✓	✓	✓
	Validation			✓	✓	✓	✓	✓	✓		✓	✓	✓	✓	✓	✓	✓
	Reporting	✓		✓	✓		✓	✓	✓		✓	✓	✓	✓	✓	✓	✓
	Implementation	✓		✓	✓	✓	✓	✓	✓		✓		✓	✓	✓		
	Surveillance	✓			✓	✓	✓		✓		✓						
**Engagement**																	
	Development	✓			✓				✓					✓			
	Validation																
	Reporting				✓									✓			✓
	Implementation								✓		✓						
	Surveillance	✓			✓												

^a^AI: artificial intelligence.

^b^CONSORT: Consolidated Standards for Reporting Trials.

^C^CAIR: Clinical AI Research.

^d^MINIMAR: Minimum Information for Medical AI Reporting.

^e^SPIRIT: Standard Protocol Items: Recommendations for Interventional Trials.

#### Transparency

Transparency describes how openly and thoroughly information is disclosed to the public and the scientific community [41]. Transparency allows for independent evaluation of an AI algorithm’s predictive power [42]. Involving stakeholders to help identify errors and bias in development or implementation also requires transparency [43]. Health care providers need transparency to interpret and justify medical decisions that result from AI use.

All but one framework (*Evaluating AI)* provided input on transparency with regard to the development and reporting of AI. Only four frameworks (*AI in Health Care, Comprehensive Checklist, Evaluating AI, 20 Critical Questions)* commented on transparency with regard to surveillance. Two frameworks (*20 Critical Questions*, *Comprehensive Checklist*) commented on transparency in regard to all five stages of translation. The number of translational stages considered for transparency ranged from 3 to 5, with an average score of 3.9 across all frameworks. On average, descriptive frameworks discussed transparency in regard to fewer stages of translation than reporting frameworks (3.5 vs 4.1).

#### Reproducibility

Reproducibility describes how likely it is that others could develop or apply an AI tool with similar results. Reproducibility is a basic tenet of good scientific practice [44]. The ability to reproduce AI models is key to external validation [45]. Reproducibility accounts for burdens such as costs and high computational needs. Reproducibility in implementation and surveillance is necessary to improve the widespread, equitable use of AI.

All frameworks commented on reproducibility. Only one (*20 Critical Questions)* commented on reproducibility in regard to all five stages of translation, and this was also the only framework to comment on reproducibility in regard to the surveillance of AI. Most frameworks described reproducibility in relation to the validation, reporting, and implementation of AI. Scores for reproducibility ranged from 1 to 5 with a mean score of 2.9 across all frameworks. On average, descriptive frameworks discussed reproducibility in regard to fewer stages of translation than reporting frameworks (2.3 vs 3.3).

#### Ethics

Ethics considers values such as benevolence, fairness, respect for autonomy, and privacy. Such values are essential to avoiding harm and ensuring societal benefit in AI use [46]. Ethical practice for the use of AI in medicine relies on collaboration with ethicists, social scientists, and regulators. Racial, gender, and insurance provider biases are the largest ethical concerns with AI use [47].

Only one framework commented on ethical considerations across all stages of translation (*Ethical Considerations)*. Four frameworks (*AI in Health Care, 20 Critical Questions,* *CAIR Checklist,* and *SPIRIT-AI)* addressed ethical considerations for development, validation, reporting, and implementation, and one tool addressed ethical considerations for development, validation, implementation, and surveillance (*Evaluating AI*). Scores for ethics ranged from 2 to 5 with a mean score of 3.4. On average, descriptive frameworks discussed ethics in regard to more stages of translation than reporting frameworks (3.7 vs 3.2).

#### Effectiveness

Effectiveness describes the success and efficiency of models and methods when they are applied in a given context. Effectiveness is concerned with matters such as data quality and model fit during the development of AI models [48]. External validation helps ensure effective discrimination and calibration to prevent overfitting [49]. Measures of effectiveness should be clearly and consistently reported [20,48]. There is a lack of appropriate benchmarks and standards of care to accurately measure the clinical benefit of many AI models [50]. Strategies are needed to continually measure effectiveness after implementation [17].

Four frameworks (*Ethical Considerations, Users’ Guide, 20 Critical Questions, Comprehensive Checklist*) commented on effectiveness across all translational stages. All frameworks reported on effectiveness as a consideration for the development of medical AI. All but one framework (*AI in Healthcare*) reported on effectiveness during validation. Six frameworks commented on effectiveness as a consideration for surveillance (*AI in Health Care, Ethical Considerations, Evaluating AI, Users’ Guide, 20 Critical Questions, Comprehensive Checklist*). Scores for effectiveness ranged from 3 to 5 with a mean score of 4.1. On average, descriptive frameworks discussed ethics in regard to more stages of translation than reporting frameworks (4.3 vs 3.9).

#### Engagement

Engagement explores to what extent the opinions and values of patients and other end users or stakeholders are collected and accounted for in decision-making. The degree of engagement can range from consultation (lowest level) to partnership and shared leadership [17]. In health research, using engagement approaches has been demonstrated to increase study enrollment, improve data quality, and improve the relevance of research design and conduct [51]. Patient engagement can also improve the quality and efficiency of health care, and reduce costs [52].

No frameworks considered engagement across all five stages. Engagement was discussed in relation to development by four frameworks (*AI in Health Care, Ethical Considerations, 20 Critical Questions, DECIDE-AI*) and in relation to reporting by three frameworks (*Ethical Considerations, DECIDE-AI, SPIRIT-AI*). No frameworks explored engagement in the validation stage of translation. Scores for engagement ranged from 0 to 3 with a mean of 0.8, which did not differ across descriptive and reporting frameworks.

## Discussion

### Principal Findings

Frameworks for applying and evaluating AI in medicine are rapidly emerging and address important considerations for the oversight of AI, such as those regarding transparency, reproducibility, ethics, and effectiveness. Providing guidance on integrating stakeholder engagement to inform AI is not a current strength of frameworks. Frameworks included in this review were the least likely to provide guidance on using engagement to inform the translation of AI in comparison to other considerations. The relative paucity of guidance on engagement reflects the larger AI landscape, which does not actively engage diverse end users in the translation of AI. For many stakeholders, AI remains a black box [53,54].

More than half of the frameworks provided reporting guidance on the use of AI in medicine. Additionally, nearly all frameworks in this review were published in 2019 or later. Given the rapid expansion of the field, it is essential to assess the consistency of recommendations across reporting frameworks to build shared understanding.

A near-miss in this review was the Transparent Reporting of a multivariable prediction model for Individual Prognosis or Diagnosis (TRIPOD) Statement [55], which provides reporting guidelines for studies using prediction models for diagnosis or prognosis. As this framework is often used to evaluate AI models, we did evaluate its content and found that it offered comments on transparency, reproducibility, and effectiveness in the translational stages of development, validation, and reporting. It also provided considerations for ethics in the validation of models, but not in other translational stages. It did not pose any guidance on the use of engagement. A TRIPOD-AI [18] extension is forthcoming, which is engaging diverse stakeholders in its development. We hope that the guidelines themselves will recommend the use of end-user engagement.

The content domains and stages of translation that we have considered are far from exhaustive, and there are many other features and specific stages of AI development, application, and evaluation that are worthy of discussion. For instance, as the scope of AI in medicine expands, it will require broadened evaluation. For instance, there have been few economic evaluations of AI tools in medicine, which may be a barrier to their implementation [56]. Another form of evaluation might include the use of randomized controlled trials to assess the efficacy of tools in clinical contexts. Another consideration is regarding conflicts of interest, and it will be important to establish approaches to evaluate and mitigate potential conflicts of interest.

None of the frameworks included in this review used an explicit translational science lens to provide explicit guidance across the AI life cycle. Having resources that detail considerations for AI application and evaluation at each stage of the translational process would be helpful for those seeking to develop AI with meaningful medical applications. Resources that could be helpful would include patient/community-centered educational resources about the value of AI, a framework to optimize the patient-centered translation of AI predictive analytics into clinical decision-making, and critical appraisal tools for use in comparing different applications of AI to inform medical decision-making.

There was a paucity of guidance regarding the surveillance of AI in medicine. Although some research has described the use of AI to inform primarily public health surveillance [57,58], little work—even outside of the frameworks included in this review—has provided specific guidance on how to surveil the use of AI with medical applications. Existing recommendations for the surveillance of pharmaceutical and other medical interventions might be applicable to AI, but tailored recommendations will also be needed. It is likely that surveillance will need to be an ongoing process to provide up-to-date information on how AI tools perform in light of new clinical information and research, and to recalibrate AI tools to incorporate this knowledge into clinical predictions [59].

The goal of the framework evaluations was not intended to reflect the *quality* of the frameworks but rather to indicate the *coverage* of AI guidance either at the individual framework level (Figure 1) or across the literature (Figure 2). These evaluations could be used as a quick reference for clinicians, developers, patients, and others to identify which framework(s) may provide the most relevant recommendations to their specific AI application. For instance, *CONSORT-AI* was specifically developed as a checklist to inform the reporting of AI research. Although it had the lowest overall score, it provided recommendations for reporting relevant to four out of the five considerations raised in this review.

The field of AI in medicine could stand to learn from the clearer methodological standards and best practices currently existent in established fields such as patient-centered outcomes research (PCOR) [51,60]. PCOR works to advance the quality and relevance of evidence about how to prevent, diagnose, treat, monitor, and manage health care; this evidence helps patients, caregivers, clinicians, policymakers, and other health care stakeholders make better decisions. The translation of AI in medicine lacks the user-centeredness that is central to PCOR [61]. At a minimum, AI for use in medicine should be developed by multidisciplinary teams, where stakeholders from relevant fields (eg, bioinformatics, specific medical specialties, patient experience) offer their expertise to inform the development of a given AI application. Ideally, more integrated transdisciplinary approaches, wherein stakeholders from relevant fields collectively create shared knowledge that transcends their individual disciplines, would be used to develop AI. Using a transdisciplinary approach has the potential to create AI that is technically robust, provides clinically relevant information, and can be easily integrated into the clinical workflow to inform patient and clinician decision-making.

### Conclusion

There is a growing literature offering input on the oversight of AI in medicine, with more guidance from regulatory bodies such as the US FDA forthcoming. Although existing frameworks provide general coverage of considerations for the oversight of AI in medicine, they fall short in their ability to offer input on the use of engagement in the development of AI, as well as in providing recommendations for the specific translational stage of surveilling AI. Frameworks should emphasize engaging patients, clinicians, and other end users in the development, use, and evaluation of AI in medicine.

## Abbreviations

AI: artificial intelligence
CONSORT: Consolidated Standards for Reporting Trials
FDA: Food and Drug Administration
PCOR: patient-centered outcomes research
SPIRIT: Standard Protocol Items: Recommendations for Interventional Trials
TRIPOD: Transparent Reporting of a multivariable prediction model for Individual Prognosis or Diagnosis
